# Supplementary material for: Changes in the relationship between attachment and emotion recognition from adolescence to adulthood
Source: PLoS One. 2025 Jun 3;20(6):e0325205. doi: 10.1371/journal.pone.0325205 (PMC12132965; doi:10.1371/journal.pone.0325205)
Supplement: S1 File — (PDF) [file pone.0325205.s001.pdf]

Neutral emotions:

4. insisting
10. cautious
11. regretful
16. thoughtful
19. tentative
27. cautious
28. interested
29. reflective
32. serious

Positive emotions:

1. playful
3. desire
6. fantasizing
13. anticipating
15. contemplative
18. decisive
20. friendly
21. fantasizing
25. interested
30. flirtatious
31. confident

Negative emotions:

2. upset
5. irritated
7. uneasy
8. despondent
9. preoccupied
12. skeptical
14. accusing
17. doubtful
22. preoccupied
23. defiant
24. pensive
26. hostile
33. concerned
34. distrustful
35. nervous
36. suspicious
