# Supplementary material for: Changes in the relationship between attachment and emotion recognition from adolescence to adulthood
Source: PLoS One. 2025 Jun 3;20(6):e0325205. doi: 10.1371/journal.pone.0325205 (PMC12132965; doi:10.1371/journal.pone.0325205)
Supplement: S2 Table — (DOCX) [file pone.0325205.s002.docx]

|  | **B** | **SE** | **β** | **t** | **p** | **VIF** | **Tolerance** |
| --- | --- | --- | --- | --- | --- | --- | --- |
| **RMETSum** | | | | | | | |
| **Step1** | | | | | | | |
| Age | 0.077 | 0.196 | 0.034 | 0.393 | 0.695 | 1.01 | 0.988 |
| Sex | 1.683 | 0.599 | 0.49 | 2.811 | 0.006 | 1.01 | 0.988 |
| **Step 2** | | | | | | | |
| Age | 0.123 | 0.194 | 0.055 | 0.636 | 0.526 | 1.03 | 0.971 |
| Sex | 1.249 | 0.621 | 0.364 | 2.013 | 0.046 | 1.13 | 0.887 |
| Avoidance | -0.898 | 0.416 | -0.192 | -2.158 | 0.033 | 1.08 | 0.925 |
| Anxiety | 0.563 | 0.311 | 0.166 | 1.813 | 0.072 | 1.16 | 0.865 |
| **RMETPos** | | | | | | | |
| **Step 1** | | | | | | | |
| Age | 0.054 | 0.103 | 0.047 | 0.52 | 0.604 | 1.01 | 0.988 |
| Sex | 0.047 | 0.315 | 0.027 | 0.149 | 0.882 | 1.01 | 0.988 |
| **Step 2** | | | | | | | |
| Age | 0.076 | 0.101 | 0.066 | 0.748 | 0.456 | 1.03 | 0.971 |
| Sex | -0.235 | 0.325 | -0.134 | -0.723 | 0.471 | 1.13 | 0.887 |
| Avoidance | -0.466 | 0.217 | -0.195 | -2.144 | 0.034 | 1.08 | 0.925 |
| Anxiety | 0.389 | 0.162 | 0.225 | 2.392 | 0.018 | 1.16 | 0.865 |
| **RMETNeg** | | | | | | | |
| **Step1** | | | | | | | |
| Age | -0.083 | 0.113 | -0.063 | -0.733 | 0.465 | 1.01 | 0.988 |
| Sex | 1.256 | 0.345 | 0.622 | 3.637 | < 0.001 | 1.01 | 0.988 |
| **Step 2** | | | | | | | |
| Age | -0.061 | 0.114 | -0.046 | -0.537 | 0.593 | 1.03 | 0.971 |
| Sex | 1.109 | 0.363 | 0.549 | 3.055 | 0.003 | 1.13 | 0.887 |
| Avoidance | -0.395 | 0.243 | -0.143 | -1.623 | 0.107 | 1.08 | 0.925 |
| Anxiety | 0.173 | 0.182 | 0.087 | 0.949 | 0.344 | 1.16 | 0.865 |
| **RMETNeut** | | | | | | | |
| **Step 1** | | | | | | | |
| Age | 0.106 | 0.088 | 0.107 | 1.21 | 0.23 | 1.01 | 0.988 |
| Sex | 0.38 | 0.269 | 0.251 | 1.41 | 0.160 | 1.01 | 0.988 |
| **Step 2** | | | | | | | |
| Age | 0.108 | 0.089 | 0.11 | 1.213 | 0.228 | 1.03 | 0.971 |
| Sex | 0.375 | 0.286 | 0.247 | 1.311 | 0.192 | 1.13 | 0.887 |
| Avoidance | -0.036 | 0.192 | -0.018 | -0.19 | 0.850 | 1.08 | 0.925 |
| Anxiety | 0.002 | 0.143 | 0.001 | 0.014 | 0.989 | 1.16 | 0.865 |
