# Supplementary material for: Changes in the relationship between attachment and emotion recognition from adolescence to adulthood
Source: PLoS One. 2025 Jun 3;20(6):e0325205. doi: 10.1371/journal.pone.0325205 (PMC12132965; doi:10.1371/journal.pone.0325205)
Supplement: S3 Table — (DOCX) [file pone.0325205.s003.docx]

|  | **B** | **SE** | **β** | **t** | **p** | **VIF** | **Tolerance** |
| --- | --- | --- | --- | --- | --- | --- | --- |
| **RMETSum - Step1** | | | | | | | |
| Age | 0.077 | 3.139 | 0.034 | 0.393 | 0.695 | 1.01 | 0.988 |
| Sex | 1.683 | 0.599 | 0.49 | 2.811 | 0.006 | 1.01 | 0.988 |
| **RMETSum - Step 2** | | | | | | | |
| Age | 0.143 | 0.191 | 0.064 | 0.748 | 0.456 | 1.08 | 0.929 |
| Sex | 0.914 | 0.606 | 0.266 | 1.508 | 0.134 | 1.16 | 0.862 |
| IRS | -0.055 | 0.41 | -0.015 | -0.133 | 0.894 | 1.82 | 0.548 |
| ADS | 0.595 | 0.487 | 0.163 | 1.221 | 0.224 | 2.64 | 0.379 |
| CF | -0.06 | 0.51 | -0.015 | -0.117 | 0.907 | 2.48 | 0.403 |
| SA | -1.536 | 0.391 | -0.389 | -3.931 | < 0.001 | 1.45 | 0.689 |
| DI | -0.308 | 0.337 | -0.093 | -0.913 | 0.363 | 1.53 | 0.653 |
| **RMETPos - Step 1** | | | | | | | |
| Age | 0.054 | 0.103 | 0.047 | 0.52 | 0.604 | 1.01 | 0.988 |
| Sex | 0.047 | 0.315 | 0.027 | 0.149 | 0.882 | 1.01 | 0.988 |
| **RMETPos - Step 2** | | | | | | | |
| Age | 0.041 | 0.099 | 0.036 | 0.416 | 0.678 | 1.08 | 0.929 |
| Sex | -0.42 | 0.316 | -0.24 | -1.332 | 0.185 | 1.16 | 0.862 |
| IRS | 0.386 | 0.213 | 0.205 | 1.811 | 0.073 | 1.82 | 0.548 |
| ADS | 0.199 | 0.254 | 0.107 | 0.783 | 0.435 | 2.64 | 0.379 |
| CF | 0.286 | 0.266 | 0.142 | 1.076 | 0.284 | 2.48 | 0.403 |
| SA | -0.716 | 0.203 | -0.355 | -3.519 | < 0.001 | 1.45 | 0.689 |
| DI | 0.25 | 0.176 | 0.148 | 1.423 | 0.157 | 1.53 | 0.653 |
| **RMETNeg - Step1** | | | | | | | |
| Age | -0.083 | 0.113 | -0.063 | -0.733 | 0.465 | 1.01 | 0.988 |
| Sex | 1.256 | 0.345 | 0.622 | 3.637 | < 0.001 | 1.01 | 0.988 |
| **RMETNeg - Step 2** | | | | | | | |
| Age | -0.034 | 0.116 | -0.026 | -0.295 | 0.005 | 1.08 | 0.929 |
| Sex | 1.063 | 0.369 | 0.527 | 2.879 | 0.005 | 1.16 | 0.862 |
| IRS | -0.077 | 0.25 | -0.036 | -0.309 | 0.758 | 1.82 | 0.548 |
| ADS | 0.052 | 0.297 | 0.024 | 0.175 | 0.861 | 2.64 | 0.379 |
| CF | -0.138 | 0.311 | -0.06 | -0.445 | 0.657 | 2.48 | 0.403 |
| SA | -0.369 | 0.238 | -0.159 | -1.55 | 0.124 | 1.45 | 0.689 |
| DI | -0.281 | 0.205 | -0.144 | -1.367 | 0.174 | 1.53 | 0.653 |
| **RMETNeut - Step 1** | | | | | | | |
| Age | 0.106 | 0.088 | 0.107 | 1.21 | 0.23 | 1.01 | 0.988 |
| Sex | 0.38 | 0.269 | 0.251 | 1.41 | 0.16 | 1.01 | 0.988 |
| **RMETNeut - Step 2** | | | | | | | |
| Age | 0.136 | 0.089 | 0.137 | 1.527 | 0.129 | 1.08 | 0.929 |
| Sex | 0.271 | 0.282 | 0.179 | 0.961 | 0.338 | 1.16 | 0.862 |
| IRS | -0.364 | 0.191 | -0.223 | -1.907 | 0.059 | 1.82 | 0.548 |
| ADS | 0.344 | 0.227 | 0.214 | 1.519 | 0.132 | 2.64 | 0.379 |
| CF | -0.207 | 0.238 | -0.119 | -0.872 | 0.385 | 2.48 | 0.403 |
| SA | -0.452 | 0.182 | -0.259 | -2.483 | 0.014 | 1.45 | 0.689 |
| DI | -0.277 | 0.157 | -0.19 | -1.765 | 0.08 | 1.53 | 0.653 |
