# Supplementary material for: Changes in the relationship between attachment and emotion recognition from adolescence to adulthood
Source: PLoS One. 2025 Jun 3;20(6):e0325205. doi: 10.1371/journal.pone.0325205 (PMC12132965; doi:10.1371/journal.pone.0325205)
Supplement: S4 Table — (DOCX) [file pone.0325205.s004.docx]

|  | **B** | **SE** | **β** | **t** | **p** | **VIF** | **Tolerance** |
| --- | --- | --- | --- | --- | --- | --- | --- |
| **RMETSum** | | | | | | | |
| **Step1** | | | | | | | |
| Age | -0.033 | 0.043 | -0.066 | -0.773 | 0.441 | 1.03 | 0.972 |
| Sex | 0.409 | 0.577 | 0.146 | 0.708 | 0.48 | 1.03 | 0.972 |
| **Step 2** | | | | | | | |
| Age | -0.053 | 0.044 | -0.107 | 0.713 | 0.23 | 1.11 | 0.902 |
| Sex | 0.419 | 0.589 | 0.15 | 0.713 | 0.477 | 1.08 | 0.929 |
| Avoidance | -0.084 | 0.359 | -0.022 | -0.235 | 0.815 | 1.24 | 0.805 |
| Anxiety | -0.436 | 0.299 | -0.139 | -1.46 | 0.147 | 1.29 | 0.774 |
| **RMETPos** | | | | | | | |
| **Step 1** | | | | | | | |
| Age | 0.014 | 0.022 | 0.054 | 0.634 | 0.527 | 1.03 | 0.972 |
| Sex | 0.119 | 0.298 | 0.083 | 0.399 | 0.69 | 1.03 | 0.972 |
| **Step 2** | | | | | | | |
| Age | <0.001 | 0.022 | 0.001 | 0.019 | 0.985 | 1.11 | 0.902 |
| Sex | 0.049 | 0.3 | 0.034 | 0.163 | 0.871 | 1.08 | 0.929 |
| Avoidance | -0.283 | 0.183 | -0.143 | -1.545 | 0.125 | 1.24 | 0.805 |
| Anxiety | -0.2 | 0.152 | -0.124 | -1.317 | 0.19 | 1.29 | 0.774 |
| **RMETNeg** | | | | | | | |
| **Step1** | | | | | | | |
| Age | -0.076 | 0.028 | -0.224 | -2.688 | 0.008 | 1.03 | 0.972 |
| Sex | 0.15 | 0.382 | 0.08 | 0.384 | 0.694 | 1.03 | 0.972 |
| **Step 2** | | | | | | | |
| Age | -0.082 | 0.029 | -0.243 | -2.793 | 0.006 | 1.11 | 0.902 |
| Sex | 0.182 | 0.392 | 0.096 | 0.463 | 0.644 | 1.08 | 0.929 |
| Avoidance | 0.055 | 0.239 | 0.021 | 0.229 | 0.819 | 1.24 | 0.805 |
| Anxiety | -0.169 | 0.199 | -0.08 | -0.851 | 0.396 | 1.29 | 0.774 |
| **RMETNeut** | | | | | | | |
| **Step 1** | | | | | | | |
| Age | 0.029 | 0.02 | 0.122 | 1.439 | 0.152 | 1.03 | 0.972 |
| Sex | 0.139 | 0.271 | 0.105 | 0.513 | 0.609 | 1.03 | 0.972 |
| **Step 2** | | | | | | | |
| Age | 0.028 | 0.021 | 0.121 | 1.362 | 0.175 | 1.11 | 0.902 |
| Sex | 0.189 | 0.279 | 0.143 | 0.678 | 0.499 | 1.08 | 0.929 |
| Avoidance | 0.144 | 0.17 | 0.079 | 0.843 | 0.401 | 1.24 | 0.805 |
| Anxiety | -0.067 | 0.141 | -0.045 | -0.469 | 0.640 | 1.29 | 0.774 |
