# Supplementary material for: Changes in the relationship between attachment and emotion recognition from adolescence to adulthood
Source: PLoS One. 2025 Jun 3;20(6):e0325205. doi: 10.1371/journal.pone.0325205 (PMC12132965; doi:10.1371/journal.pone.0325205)
Supplement: S5 Table — (DOCX) [file pone.0325205.s005.docx]

|  | **B** | **SE** | **β** | **t** | **p** | **VIF** | **Tolerance** |
| --- | --- | --- | --- | --- | --- | --- | --- |
| **RMETSum - Step1** | | | | | | | |
| Age | -0.033 | 0.043 | -0.066 | -0.773 | 0.441 | 1.03 | 0.972 |
| Sex | 0.409 | 0.577 | 0.146 | 0.708 | 0.48 | 1.03 | 0.972 |
| **RMETSum - Step 2** | | | | | | | |
| Age | -0.051 | 0.045 | -0.102 | -1.131 | 0.26 | 1.16 | 0.860 |
| Sex | 0.628 | 0.609 | 0.225 | 1.032 | 0.304 | 1.15 | 0.869 |
| IRS | -0.38 | 0.375 | -0.106 | 1.013 | 0.313 | 1.56 | 0.639 |
| ADS | -0.6 | 0.4 | -0.2 | -1.506 | 0.134 | 2.47 | 0.404 |
| CF | -0.216 | 0.45 | -0.064 | -0.481 | 0.631 | 2.52 | 0.397 |
| SA | 0.294 | 0.362 | 0.078 | 0.812 | 0.418 | 1.30 | 0.769 |
| DI | 0.206 | 0.25 | 0.079 | 0.828 | 0.409 | 1.28 | 0.781 |
| **RMETPos - Step 1** | | | | | | | |
| Age | 0.014 | 0.022 | 0.054 | 0.634 | 0.527 | 1.03 | 0.972 |
| Sex | 0.12 | 0.298 | 0.083 | 0.399 | 0.69 | 1.03 | 0.972 |
| **RMETPos - Step 2** | | | | | | | |
| Age | 0.005 | 0.022 | 0.021 | 0.242 | 0.809 | 1.16 | 0.860 |
| Sex | 0.155 | 0.301 | 0.108 | 0.515 | 0.607 | 1.15 | 0.869 |
| IRS | -0.134 | 0.185 | -0.073 | -0.723 | 0.471 | 1.56 | 0.639 |
| ADS | -0.671 | 0.196 | -0.433 | -3.416 | <0.001 | 2.47 | 0.404 |
| CF | -0.292 | 0.222 | -0.168 | -1.314 | 0.191 | 2.52 | 0.397 |
| SA | 0.013 | 0.179 | 0.007 | 0.072 | 0.943 | 1.3 | 0.769 |
| DI | 0.192 | 0.123 | 0.142 | 1.558 | 0.122 | 1.28 | 0.781 |
| **RMETNeg - Step1** | | | | | | | |
| Age | -0.076 | 0.028 | -0.224 | -2.688 | 0.008 | 1.03 | 0.972 |
| Sex | 0.15 | 0.382 | 0.08 | 0.394 | 0.694 | 1.03 | 0.972 |
| **RMETNeg - Step 2** | | | | | | | |
| Age | -0.08 | 0.03 | -0.236 | -2.635 | 0.009 | 1.16 | 0.86 |
| Sex | 0.263 | 0.407 | 0.139 | 0.646 | 0.519 | 1.15 | 0.869 |
| IRS | -0.107 | 0.251 | -0.044 | -0.427 | 0.67 | 1.56 | 0.639 |
| ADS | -0.283 | 0.266 | -0.139 | -1.062 | 0.29 | 2.47 | 0.404 |
| CF | -0.144 | 0.301 | -0.063 | -0.477 | 0.634 | 2.52 | 0.397 |
| SA | 0.129 | 0.243 | 0.05 | 0.531 | 0.596 | 1.30 | 0.769 |
| DI | 0.137 | 0.167 | 0.077 | 0.82 | 0.414 | 1.28 | 0.781 |
| **RMETNeut - Step 1** | | | | | | | |
| Age | 0.029 | 0.02 | 0.122 | 1.439 | 0.152 | 1.03 | 0.972 |
| Sex | 0.139 | 0.271 | 0.105 | 0.513 | 0.609 | 1.03 | 0.972 |
| **RMETNeut - Step 2** | | | | | | | |
| Age | 0.023 | 0.021 | 0.099 | 1.092 | 0.277 | 1.16 | 0.86 |
| Sex | 0.21 | 0.286 | 0.159 | 0.734 | 0.464 | 1.15 | 0.869 |
| IRS | -0.139 | 0.176 | -0.082 | -0.787 | 0.433 | 1.56 | 0.639 |
| ADS | 0.355 | 0.187 | 0.25 | 1.901 | 0.059 | 2.47 | 0.404 |
| CF | 0.219 | 0.211 | 0.138 | 1.039 | 0.301 | 2.52 | 0.397 |
| SA | 0.153 | 0.17 | 0.086 | 0.897 | 0.371 | 1.3 | 0.769 |
| DI | -0.123 | 0.117 | -0.099 | -1.045 | 0.298 | 1.28 | 0.781 |
