# Supplementary material for: Changes in the relationship between attachment and emotion recognition from adolescence to adulthood
Source: PLoS One. 2025 Jun 3;20(6):e0325205. doi: 10.1371/journal.pone.0325205 (PMC12132965; doi:10.1371/journal.pone.0325205)
Supplement: S6 Table — (DOCX) [file pone.0325205.s006.docx]

|  | **18-23 years** | **24-29 years** | **30-35 years** | **36-41 years** | **42-50 years** |
| --- | --- | --- | --- | --- | --- |
| **Males** | 22 | 4 | 2 | 0 | 3 |
| **Females** | 88 | 20 | 1 | 0 | 2 |
