# Supplementary material for: Changes in the relationship between attachment and emotion recognition from adolescence to adulthood
Source: PLoS One. 2025 Jun 3;20(6):e0325205. doi: 10.1371/journal.pone.0325205 (PMC12132965; doi:10.1371/journal.pone.0325205)
Supplement: Attachment — Submitted filename: response to reviewers.docx [file pone.0325205.s008.docx]

**Responses to Reviewer 1**

**In the abstract, “we present findings …” should be rephrased as the study is not just on “young adults and adolescents” as the age range is up to 50 years old.**

Thank you for the suggestion, the adjustment has been made.

**In my opinion, the introduction is too long and expands too much about early childhood and infants which makes it confusing since it is not the purpose of the study. But I leave that to the author and editor to decide whether to shorten it.**

I agree with the reviewer that the first part of the introduction was lengthy, therefore I have shortened it somewhat (see paragraph 1). However, in order to shed some light on how attachment may play a role in adult theory of mind and emotion recognition specifically, it is important to understand the mechanisms that may contribute to this potential link. Since attachment characteristics are rooted in early experiences, I believe it is necessary to discuss these potential mechanisms from a developmental point of view. Therefore, the sections that present how biases in information processing may appear as a result of attachment insecurity have not been deleted or shortened.

**Did the author consider the interaction between age and sex as a predictor (and if not, why not?)? Especially in the adult group.**

I thank the reviewer for the suggestion. Indeed, interaction terms would be interesting to add to the analyses; however, it is unfortunately not possible with the current dataset as detecting interactions in the regression models requires a significantly larger sample size (for discussions on this see for example *Leon, A. C., & Heo, M. (2009). Sample Sizes Required to Detect Interactions between Two Binary Fixed-Effects in a Mixed-Effects Linear Regression Model. Computational statistics & data analysis, 53(3), 603–608.* [*https://doi.org/10.1016/j.csda.2008.06.010*](https://doi.org/10.1016/j.csda.2008.06.010) or https://statmodeling.stat.columbia.edu/2018/03/15/need16/

). In addition, I have run a power analysis for the interaction term with standard parameters (effects size: 0.2; alpha=0.05.; power=0.8, 1 covariate (age) and 2 groups (for sex)), and it resulted in a required sample size of 416 which is signficantly higher than my own. Thus, this particular study is underpowered to reliably test these effect. Nonetheless, I trust that in spite of this, the reviewer will find that the current results are still sound enough to publish - with acknowledging the limitations, of course. To ensure clarity on this issue, I have extended the discussion (see next point also).

I would also like to point out that although the age range is rather wide, the majority of the participants fall at the lower end of the age scale (see also below).

***Also, there is a large discrepancy in sample size between males and females in the adult group. Could that reflect a bias in recruitment? This should be acknowledged as a limitation, or discussed as a potential explanation of differences between the adolescents and adults.***

Indeed, recruitment differed for the adult and the adolescent samples. Adolescents were recruited in high schools and were tested in their own classrooms, thus gender distribution here largely resembles that of the classes themselves. Adults, on the other hand, were recruited through a university course for credit. These participants were thus university students. Although recruitment did not limit the faculties from which students may have applied (with the only exception that psychology students could only participate if they were in their freshman year), with this method of recruitment, we generally end up with an uneven distribution of females and males. The discussion has been extended to reflect on this issue (lines 555-561.).

***In this context, was age correlated with the different variables tested? A supplementary Figure displaying the distribution of sex by age might be informative.***

Thank you for the suggestion, another Supplemantary file (S6) have been added to show the distribution of participants by age and gender. Moreover, both for adolescents and adults zero-order correlations involving age have been calculated and reported in the preliminary analysis sections (lines 270-271 and 400-403).

***Is there any information about mental health / neurodevelopmental disorders for the participants? Were any participants autistic (for instance) as it could significantly impact their capacity to identify emotions. This information should be clarified.***

During recruitment, we explicitly stated that a lack of any psychiatric diagnosis was a precondition for participation. Since it would be difficult to unambiguously define conditions that do not affect such social cognitive skills, we felt it was necessary to extend this constraint to any diagnosis. A sentence has been added to the methods section to make this explicit in the manuscript (lines 192-193).

***In S1_File, in the neutral emotion section, cautious is listed twice.***

Some of the expressions may appear multiple times in the file since the classification for valence was done on each item by presenting both the verbal expression and the picture. Since there are some items in the RMET with the same emotion expression as the correct choice, these appear separately in this document.

**Response to Reviewer 2**

I am grateful for the kind words on our work and the manuscript.
